# Effect of povidone-iodine and propanol-based mecetronium ethyl sulphate on antimicrobial resistance and virulence in *Staphylococcus aureus*

**DOI:** 10.1186/s13756-022-01178-9

**Published:** 2022-11-11

**Authors:** Nada A. Barakat, Salwa A. Rasmy, Alaa El-Dien M. S. Hosny, Mona T. Kashef

**Affiliations:** Department of Microbiology and Immunology, Faculty of Pharmacy, Cairo University, Cairo, 11562 Egypt

**Keywords:** Antibiotics, Biocides, Biofilm, Hemolysin, Povidone-iodine, Propanol-based mecetronium ethyl sulphate, Resistance, *Staphylococcus aureus*, Virulence

## Abstract

**Background:**

Reports are available on cross-resistance between antibiotics and biocides. We evaluated the effect of povidone-iodine (PVP-I) and propanol-based mecetronium ethyl sulphate (PBM) on resistance development, antibiotics cross-resistance, and virulence in *Staphylococcus aureus*.

**Methods:**

The minimum inhibitory concentration (MIC) of PVP-I and PBM were determined against *S. aureus* ATCC 25923 using the agar-dilution method. *Staphylococcus aureus* ATCC 25923 was subjected to subinhibitory concentrations of the tested biocides in ten consecutive passages followed by five passages in a biocide-free medium; MIC was determined after each passage and after the fifth passage in the biocide-free medium. The developed resistant mutant was tested for cross-resistance to different antibiotics using Kirby-Bauer disk diffusion method. Antibiotic susceptibility profiles as well as biocides’ MIC were determined for 97 clinical *S. aureus* isolates. Isolates were categorized into susceptible and resistant to the tested biocides based on MIC distribution pattern. The virulence of the biocide-resistant mutant and the effect of subinhibitory concentrations of biocides on virulence (biofilm formation, hemolysin activity, and expression of virulence-related genes) were tested.

**Results:**

PVP-I and PBM MIC were 5000 μg/mL and 664 μg/mL. No resistance developed to PVP-I but a 128-fold increase in PBM MIC was recorded, by repeated exposure. The developed PBM-resistant mutant acquired resistance to penicillin, cefoxitin, and ciprofloxacin. No clinical isolates were PVP-I-resistant while 48.5% were PBM-resistant. PBM-resistant isolates were more significantly detected among multidrug-resistant isolates. PVP-I subinhibitory concentrations (¼ and ½ of MIC) completely inhibited biofilm formation and significantly reduced hemolysin activity (7% and 0.28%, respectively). However, subinhibitory concentrations of PBM caused moderate reduction in biofilm activity and non-significant reduction in hemolysin activity. The ½ MIC of PVP-I significantly reduced the expression of *hla*, *ebps*, *eno*, *fib*, *ica*A, and *ica*D genes. The virulence of the biocide-resistant mutant was similar to that of parent strain.

**Conclusion:**

PVP-I is a highly recommended antiseptic for use in healthcare settings to control the evolution of high-risk clones. Exposure to PVP-I causes no resistance-development risk in *S. aureus,* with virulence inhibition by subinhibitory concentrations. Also, special protocols need to be followed during PBM use in hospitals to avoid the selection of resistant strains.

**Supplementary Information:**

The online version contains supplementary material available at 10.1186/s13756-022-01178-9.

## Background

Antimicrobial resistance is one of the global threats in all healthcare sectors; the emergence of antimicrobial resistance is associated with high morbidity and mortality rates. According to the centers for disease control and prevention (CDC), bacterial resistance to antibiotics causes thousands of deaths per year and an increase in hospitalization length worldwide [1]. The World Health Organization predicted that by year 2050 ten million people will die yearly due to antimicrobial resistance [2]. The improper use and over-prescription of antibiotics have been considered the driving factors of the emergence of microbial resistance. However, the role of non-antibiotic antimicrobials in resistance development has been ignored for years [3, 4].

Non-antibiotic antimicrobials (antiseptics, disinfectants, and preservatives) are extensively used in healthcare settings to control infections and microbial contamination [5]; they are used for general equipment sterilization, disinfection of hospital surfaces and pre-operative skin decontamination of patients and staff [6]. The indiscriminate use and repeated exposure to these agents, over a long time, can select resistant strains and cause antibiotic cross-resistance [7, 8]; for example, the exposure to chlorhexidine led to reduced susceptibility to penicillin, gentamicin and tetracycline in *Staphylococcus aureus* clinical strains [9]*.* Also, benzalkonium chloride-adapted *Pseudomonas aeruginosa* showed cross- resistance to polymyxin [10]. Repeated exposure of *P. aeruginosa* to chlorhexidine caused chlorhexidine resistance with cross-resistance to different antibiotics [11]. The U.S. Food and Drug Administration (FDA) suspended the use of triclosan in soap preparations because of its ability to trigger antibiotic cross-resistance [12].

*S. aureus* is one of the most popular nosocomial human pathogens that causes different types of diseases, ranging from skin infections to serious life-threatening infections such as bone, bloodstream, and soft tissue infections [13]. According to CDC, most hospitalized patients in intensive care units and patients with weakened immune systems or chronic conditions are at high risk of developing serious *S. aureus* infections [14]. *S. aureus* can survive on inanimate surfaces such as hospital floors or door handles even after disinfection and can be transmitted causing various ranges of serious infections [15].

*S. aureus* secrets many virulence factors including the alpha-hemolysin toxin encoded by *hla* gene which is considered the most common pore-forming toxin produced by *S. aureus*; it plays a marked role in pathogenesis [16]. The formation of biofilm offers a protective barrier against host-immune response and antimicrobials effect, thus complicating the treatment of *S. aureus* infections [17]. Biofilm production and accumulation are mediated by microbial surface components recognizing adhesive matrix molecules (MSCRAMMs), encoded by many genes including *eno*, *fib, epbs*. MSCRAMMs are a group of surface exposed proteins that can bind to host extracellular matrix factors such as elastin, laminin, and fibrinogen [13]. Polysaccharide intracellular adhesion (PIA) proteins; encoded by *ica*A and *ica*D genes are also necessary for biofilm production [13].

Treatment of *S. aureus* infections became progressively more complicated due to the emergence of antibiotic resistance. More than 90% of *S. aureus* isolates were resistant to penicillin by the early 2000s [18]. Additionally, methicillin resistant *S. aureus* (MRSA) has been listed as a serious threat by CDC [19]. Vancomycin remained the drug of choice to tackle MRSA, but the emergence of vancomycin resistance limited its clinical utility and necessitates the development of new antibiotics [18]. Inhibition of virulence in *S. aureus* infections enables the host-immune system to overcome the infective agents [20].

Povidone-iodine (PVP-I) and propanol-based mecetronium ethyl sulphate (PBM) are among the most extensively used antiseptics in hospitals [21–23]. PVP-I is known for its broad-spectrum activity and has been proven to be effective as a pre-operative surgical scrub and in preventing post-operative wound infections while PBM is used as a hand disinfectant due to its well-known antimicrobial properties and affordable price [23, 24]. No resistance to PVP-I was documented to date [21]; however, no studies are available on possible resistance development to PBM.

Here, we evaluated the possible resistance development to PVP-I and PBM and antibiotic cross-resistance, by repeated exposure in a standard *S. aureus* strain and in clinical *S. aureus* isolates from an Egyptian hospital that was using PVP-I and PBM during the time of isolates’ collection. Additionally, we assessed the effect of subinhibitory concentrations of these biocides on *S. aureus* virulence.

## Methods

### Bacterial strains, media and growth conditions

*S. aureus* ATCC 25923 was used as a reference strain. Clinical isolates (n = 97) of *S. aureus* were collected from El-Kasr El-Aini hospital, Cairo, Egypt between the period of June 2018 and August 2019; they were from both hospitalized (n = 46) and non-hospitalized patients (n = 51) as indicated in Additional file 1: Table S1. The collected isolates were from different specimens; wound (n = 44), blood (n = 34), sputum (n = 8), pus (n = 5), bronchial lavage (n = 2), urine (n = 1), burn (n = 1), aspiration fluid (n = 1) and ulcer swab (n = 1). *S. aureus* isolates were identified phenotypically according to Bergey’s Manual For Determinative Bacteriology [25] and confirmed by DNase test and tube coagulase test [26]. Isolates were preserved in nutrient broth (NB; Oxoid, England) containing 30% glycerol at -80 °C. When needed and unless otherwise described, bacteria from glycerol stock were retrieved by isolation on Muller-Hinton (MH) agar (Himedia, India) followed by overnight incubation at 37 °C.

### Biocides and antimicrobial agents

Two biocides were tested in this study using their commercially available preparations; PVP-I (betadine^®^; Nile Pharm. and Chem. Ind. Co., Egypt) containing 10% w/v povidone-iodine and PBM (sterillium^®^; BODE Chemie, Germany) containing 45% w/w 1- propanol, 30% w/w 2-propanol, and 0.2% w/w mecetronium ethyl sulphate. These products were used in the hospital during the time of collection of the clinical isolates.

The following antimicrobial agents were used for antimicrobial susceptibility testing; penicillin (10 µg), gentamicin (10 µg), erythromycin (15 µg), tetracycline (30 µg), ciprofloxacin (5 µg), clindamycin (2 µg), sulfamethoxazole/trimethoprim (1.25/23.75 µg), chloramphenicol (30 µg), rifampin (5 µg), linezolid (30 µg) and cefoxitin (30 µg). All were used as disks and were purchased from Oxoid, England. Vancomycin powder was from Sigma tech, Egypt.

### Determination of biocides minimum inhibitory concentration (MIC)

The MIC of PVP-I and PBM were determined using the agar dilution method according to the Clinical and Laboratory Standards Institute (CLSI) guidelines [27]. PVP-I and PBM were tested in a concentration range from 312.5 to 10,000 μg/mL and 332 to 85,000 μg/mL, respectively. Briefly, overnight culture of *S. aureus* ATCC 25923 in MH broth (Himedia, India) was diluted to an optical density (OD) equivalent to that of 0.5 McFarland standard (≈1  × 10^8^ CFU/mL) followed by further 1:10 dilution. The diluted culture was spotted (2 μL/spot, ≈ 10^4^ CFU) on the surface of the MH agar plates containing different concentrations of the biocides. After 24 h of incubation at 37 °C, the MIC was determined as the lowest concentration of the tested biocide that completely inhibited the growth of the organism.

### Generation of biocides’ resistant mutant

Biocides’ resistant mutants were generated by repeated exposure to a subinhibitory concentrations of the tested biocides (PVP-I or PBM) for ten consecutive passages. The method was adopted from Winder et al. [28] with minor modifications. Briefly, overnight culture of *S. aureus* ATCC 25923 was diluted to have turbidity equivalent to that of 0.5 McFarland standard and 100 µL of the diluted culture was used to inoculate 10 mL MH broth containing ½ of MIC of the tested biocide (Passage 1). After incubation at 37 °C for 24 h, the biocides’ MIC of the resulting culture from passage 1 (P1) was determined as described before. Then, 100 μL of P1 culture was used to inoculate 10 mL MH broth containing ½ of MIC of P1 for each biocide. This procedure was repeated for ten successive passages. To ensure the stability of the biocide resistant strain, cells from P10 were serially passaged in biocide-free medium for five successive passages; the MIC was determined at the end of the 5th passage, as described previously. Cultures that failed to restore the initial susceptibility in the absence of the biocides were considered as biocide-resistant mutants.

### Antibiotic susceptibility testing

The antibiotic susceptibility profiles of the parent *S. aureus* ATCC 25923 and the developed biocide-resistant mutant were determined to assess the possible cross-resistance between the tested biocide and different antibiotics. Antibiotic susceptibility testing was carried out using the Kirby-Bauer disk diffusion method according to the CLSI guidelines [29]. In brief, overnight cultures of both the parent *S. aureus* ATCC 25923 and the biocide-resistant mutant in MH broth were diluted to reach an OD equivalent to that of 0.5 McFarland standard. The adjusted bacterial suspension was distributed evenly using a sterile cotton swab on the surface of a MH agar plate and allowed to dry for five min; after which antibiotic disks were dispensed onto the surface of the agar plate. Agar plates were incubated for 24 h at 37 °C. After incubation, inhibition zones’ diameters around antibiotic disks were measured, and the results were interpreted according to CLSI breakpoints [30]. Cefoxitin was used as a methicillin surrogate to identify MRSA following CLSI recommendations [30].

The susceptibility of *S. aureus* to vancomycin was determined by broth microdilution method according to CLSI recommendations [27]. Vancomycin stock solution was prepared as 1280 μg/mL. Two-fold serial dilutions of vancomycin were made in 50 µL MH broth to obtain final concentration range between 0.5- 64 μg/mL. The overnight culture of *S. aureus* was adjusted to have an OD equivalent to that of 0.5 McFarland standard. *S. aureus* was further diluted (1:150) in MH broth (≈ 10^6^ CFU/mL) and the diluted culture (50 µL) was added to each well so that the final inoculum concentration is 5 × 10^5^ CFU/mL. After 24 h of incubation at 37 °C, the MIC was determined as the lowest concentration of vancomycin that completely inhibited the growth of the organism. Results were interpreted according to CLSI breakpoints [30].

### Susceptibility of *S. aureus* clinical isolates to the biocides and different antibiotics

The MIC of PVP-I and PBM were determined for the clinical *S. aureus* isolates as previously described. Since no CLSI breakpoints are available for interpretation of the antimicrobial susceptibility of isolates to biocides, we employed the definition of the epidemiological cut-off value for determination of resistant isolates, as suggested previously [31], where resistant isolates were defined as isolates with MIC > the epidemiological cut-off value. In natural isolates, the epidemiological cut-off value is the concentration representing 99.9% of the isolates MIC (MIC_99.9_), in isolates with unimodal MIC distribution. If the distribution of MIC is bimodal, the epidemiological cut-off value is the MIC between the two main populations. Natural isolates are defined as the isolates collected from any source even from infections and not subjected to multiple subcultures under laboratory conditions [32], where all the collected isolates in this study were considered natural.

The antibiotic susceptibility profile of the clinical *S. aureus* isolates was determined, as previously described. MRSA were the *S. aureus* strains that were cefoxitin resistant. Multidrug resistant (MDR) isolates were identified according to the definition of Magiorakos et al. [33] as being resistant to at least one agent in three or more antimicrobial categories or were MRSA.

### Assessment of virulence

The virulence of the generated biocide-resistant strain as well as of the parent *S. aureus* ATCC 25923 under the effect subinhibitory concentrations (¼ and ½ of MIC) of each tested biocide was evaluated.

### Growth curve determination

The growth patterns of the parent *S. aureus* ATCC 25923 in the absence and presence of ¼ and ½ of the MIC of each tested biocide (1250 & 2500 μg/mL, respectively for PVP-I and 166 & 332 μg/mL, respectively for PBM) as well as the growth pattern of the developed biocide-resistant strain (P 15) were determined. Briefly, the parent *S. aureus* ATCC 25923 and the developed biocide-resistant strain were grown in Tryptone Soya broth (TSB; HiMedia, India) with shaking at 200 rpm at 37 °C for 24 h. Aliquots (1 ml) of the resulting culture of the parent *S. aureus* ATCC 25923 were inoculated into 50 mL TSB either in the absence or in presence of ¼ or ½ of MIC of the tested biocide. The developed biocide-resistant strain was inoculated in TSB only. Uncultured TSB was used as a negative control. Cultures were incubated at 37 °C with shaking at 200 rpm for 24 h; the OD of the cultures at 600 nm was measured hourly for 5 h then after 24 h. The experiment was performed in triplicates and the growth curve was constructed by plotting the mean optical density against time.

### Quantitative determination of biofilm formation

Biofilm formation was assayed using the tissue culture plate method according to Christensen et al. [34]. Briefly, the tested strains (the parent *S. aureus* ATCC 25923 and the developed biocide-resistant strain; P15) were grown in TSB at 37 °C for 24 h with shaking at 180 rpm then diluted (1: 100) in TSB supplemented with 1% (w/v) glucose (AVI-CHEM LABORATORIES, India). In the case of testing biofilm formation in presence of ¼ and ½ of MIC of the biocides, TSB contained the corresponding biocide concentration. Diluted cultures (200 µL) were transferred to a flat-bottomed 96-well microtiter plate. Uncultured TSB was used as a negative control. Plates were incubated for 24 h at 37 °C under static conditions. Bacterial growth was measured at 600 nm using a microtiter plate reader (BioTek, USA) then the content of each well was decanted to remove non-adherent cells and washed three times with sterile phosphate-buffered saline (PBS; PAN-Biotech, Germany). Adherent biofilm cells were fixed using 95% ethanol (PIOCHEM, Egypt) for 20 min and then rinsed by flicking. Adherent cells were stained with 1% crystal violet for 30 min. The unbound stain was removed by washing three times with sterile distilled water. The plate was allowed to air-dry and then eluted in 95% ethanol for 15 min. The OD of the stained biofilms was measured at 570 nm using a microtiter plate reader. The assay was performed in triplicates. Biofilms’ OD were normalized to the growth OD at 600 nm. The mean normalized OD was calculated for each test condition and the cut-off value (ODc) was established as the mean OD of negative control + 3*SD. The tested strains were categorized according to the following criteria:

- Non-biofilm producer: OD ≤ ODc
- Weak biofilm producer: ODc < OD ≤ 2*ODc
- Moderate biofilm producer: 2*ODc < OD ≤ 4*ODc
- Strong biofilm producer: 4*ODc < OD

### Assay of alpha-hemolysin activity

The hemolytic action of alpha-hemolysin was measured in the parent *S. aureus* ATCC 25923 in the absence and presence of ¼ and ½ of MIC of either PVP-I or PBM as well as in the developed biocide-resistant strain (P15), according to Duan et al. [16] with minor modifications. Cultures of the tested strain in TSB were grown overnight at 37 °C with shaking at 200 rpm. Aliquots (1 mL) of the overnight culture of *S. aureus* ATCC 25923 were inoculated in 50 mL TSB either in the absence or in presence of ¼ or ½ of MIC of the tested biocides. The developed biocide-resistant strain was inoculated in TSB only. All cultures were incubated with shaking at 200 rpm overnight to reach the post-exponential phase (OD_600_ = 2.5). Bacterial cultures were then centrifuged at 5500×*g* for 2 min at 4 °C. The supernatant (100 µL) was added to 25 μL of defibrinated fresh rabbit blood and 875 μL sterile PBS and incubated at 37 °C for 1 h then centrifuged at 5500×*g* for 1 min at room temperature. The OD of the supernatant was measured at 543 nm. Sterile 1% triton X-100 (Sigma Aldrich, United States) and sterile PBS were used as positive and negative controls, respectively. The assay was performed in triplicates. The hemolytic percentage was calculated according to the following formula.

Hemolysis percentage = [(OD (test) − OD (negative control)/(OD (positive control) − OD (negative control)] × 100.

### Quantitative determination of gene expression

Quantitative real-time polymerase chain reaction (qRT-PCR) was performed to measure the changes in transcriptional levels of virulence genes; *hla, epbs, eno, fib, ica*A*,* and *ica*D in the presence of a subinhibitory concentration of PVP-I. Primers were designed using PrimerQuest Tool of integrated DNA technologies (IDTDNA, United states). *16S rRNA* gene was used as a house-keeping gene. All primers were purchased from Macrogen, South Korea, and their sequences are listed in Table 1. Overnight culture of *S. aureus* ATCC 25923 was diluted in TSB in the absence (control; C) and in the presence (Treatment; T) of ½ of MIC of PVP-I then incubated at 37 °C with shaking at 200 rpm for 2.5 h (mid-log phase, OD_600_ = 0.3) [35]. Total RNA was extracted using QIAamp RNeasy Mini kit (Qiagen, Germany,GmbH) according to the manufacturer’s instructions. On column digestion was done using RNase-free DNase 1(Qiagen, Germany) to remove residual DNA according to the manufacturer’s directions. RNA quantity and integrity were measured using a spectrophotometer at 260 nm.

**Table 1:** Sequences of primers used in the study

Target gene	Primers	Sequence (5′ → 3′)	Reference
*hla*	hla-F	GGTACCATTGCTGGTCAGTATAG	This study
	hla-R	GCAACTGTACCTTAAATGCTGAAG	
*epbs*	epbs-F	GGTGCAATGGGTGTTTCTAAAG	
	epbs-R	CGCTTTATCCTCAGTCGAGTTAT	
*eno*	eno-F	TGGTTACAAACCAGGTGAAGAA	
	eno-R	CGCCTTCGAACTTACTGTAGTC	
*fib*	fib-F	CTTGTTCAAACGTACAAGAACTG	
	fib-R	TCATAGCTGTGTTGGAAATGAA	
*icaA*	icaA-F	CATCAATCGTATTGCCAGGTAAAT	
	icaA-R	GTAAGCTCGCTGCCCTATTA	
*icaD*	icaD-F	AGCCCAGACAGAGGGAATA	
	icaD-R	ACGATATAGCGATAAGTGCTGTT	
*16S rRNA*	16S rRNA-F	GTGGAGGGTCATTGGAAACT	
	16S rRNA- R	CACTGGTGTTCCTCCATATCTC	

qRT-PCR was performed in a Stratagene MX3005P real-time PCR machine, using QuantiTect SYBR Green PCR Master Mix (Qiagen, Germany, GmbH) following the manufacturer’s recommended protocol, using the same starting RNA amount. Amplification curves and cycle threshold (Ct) values were determined by the stratagene MX3005P software. The Ct of each sample was normalized with that of the house-keeping gene and compared to that of the control.

### Statistical analysis

Experimental data were analyzed using GraphPad Prism software (version 8.0.1, La Jolla, CA, United States). The Chi-square test was used to evaluate the distribution of MRSA and Methicillin susceptible *S. aureus* (MSSA), MDR and non-MDR isolates among isolates with different biocide-resistance patterns as well as the distribution of different biocides-resistance patterns among isolates from hospitalized and non-hospitalized patients. One-way ANOVA and Dunnett’s test (post hoc test) were used to compare the results of biofilm and hemolysin assays to those of *S. aureus* cultured in the biocide-free medium as a control. The results of qRT-PCR were analyzed using two-way ANOVA compared to *S. aureus* ATCC 25923 results using Bonferroni’s test as a post hoc test. *p*-values < 0.05 were considered statistically significant.

## Results

### Biocides’ MIC of *S. aureus* ATCC 25923

The MIC of PVP-I and PBM against *S. aureus* ATCC 25923 was 5000 μg/mL and 664 μg/mL, respectively.

### Generation of biocides’ resistant mutants

**Fig. 1:**
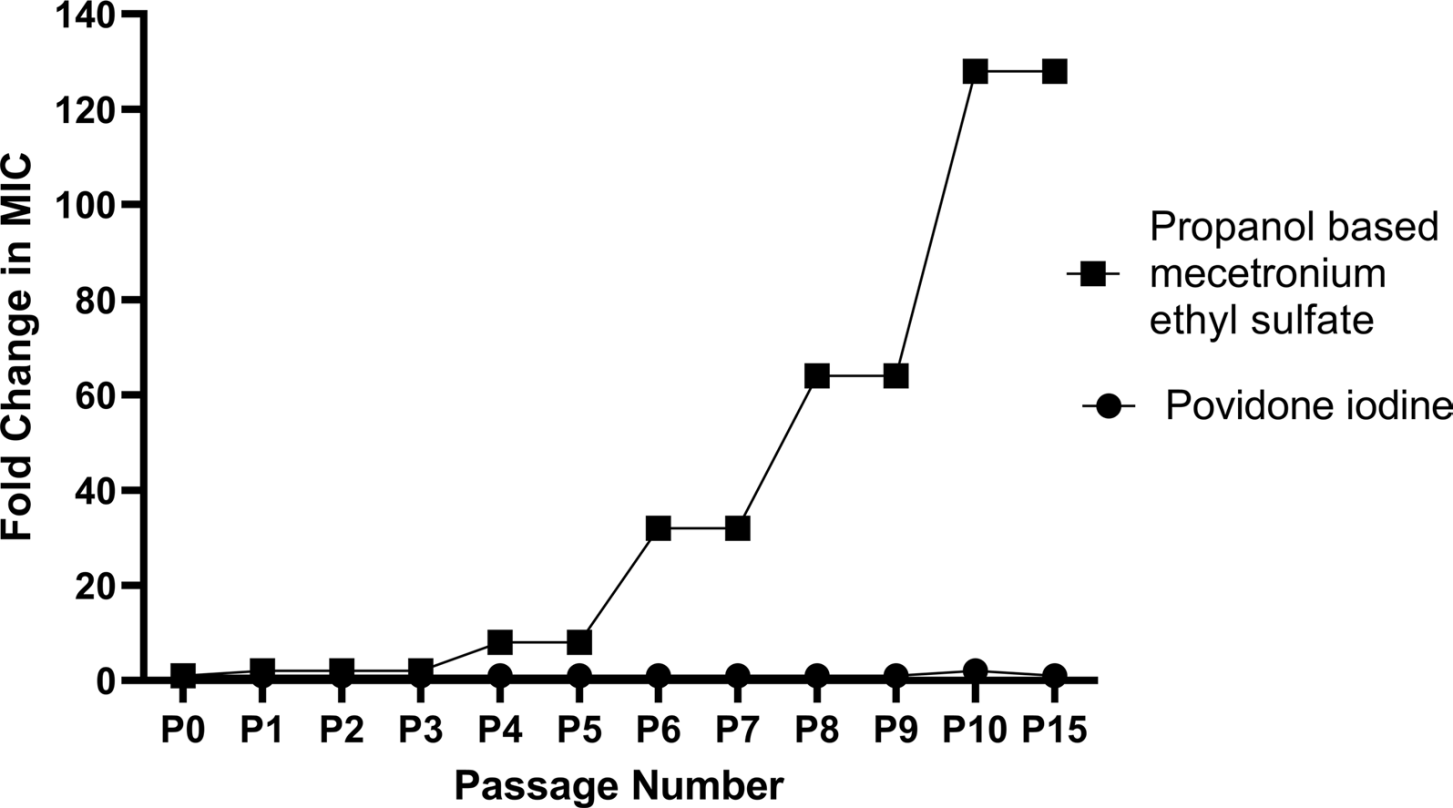
Development of biocide-resistant mutants. Fold change in the minimum inhibitory concentration (MIC) of *Staphylococcus aureus* ATCC 25923 (P0) after passage in subinhibitory concentrations of the tested biocides (Povidone-iodine and propanol-based mecetronium ethyl sulphate) for ten consecutive passages (P1–P10) and after five passages in biocide-free medium (P15)

Exposure of *S. aureus* ATCC 25923 to subinhibitory concentrations of PVP-I for ten consecutive passages resulted in only a two-fold increase in MIC from 5000 μg/mL in the parent strain to 10,000 μg/mL in P10 which reverted to the original MIC of 5000 μg/mL when passaged consecutively in biocide-free medium (Fig. 1). However, exposure to subinhibitory concentration of PBM for ten consecutive passages resulted in a gradual increase in MIC from 664 μg/mL in parent strain to 85,000 μg/mL after ten passage (increased by 128-fold). Passage of the resultant resistant strain (P10) in the PBM-free medium for five successive passages maintained the same MIC value of P10 (85,000 μg/mL for P15; Fig. 1). The developed PBM-resistant mutant was used for further study.

### Antimicrobial susceptibility profile of the parent *S. aureus* ATCC 25923 and PBM-resistant mutant

The antibiotic susceptibility profiles of the parent *S. aureus* ATCC 25923 and the PBM-resistant mutant were determined using the Kirby-Bauer disk diffusion method. *S. aureus* ATCC 25923 was sensitive to all the tested antibiotics. The PBM-resistant mutant acquired cross-resistance to penicillin, cefoxitin, and ciprofloxacin, and intermediate resistance level to clindamycin, compared to the parent strain. The PBM-resistant mutant maintained the susceptibility to vancomycin, but vancomycin MIC increased by four-fold compared to the parent strain (Additional file 1).

### Susceptibility to biocides and antimicrobials in clinical *S. aureus* isolates

**Fig. 2:**
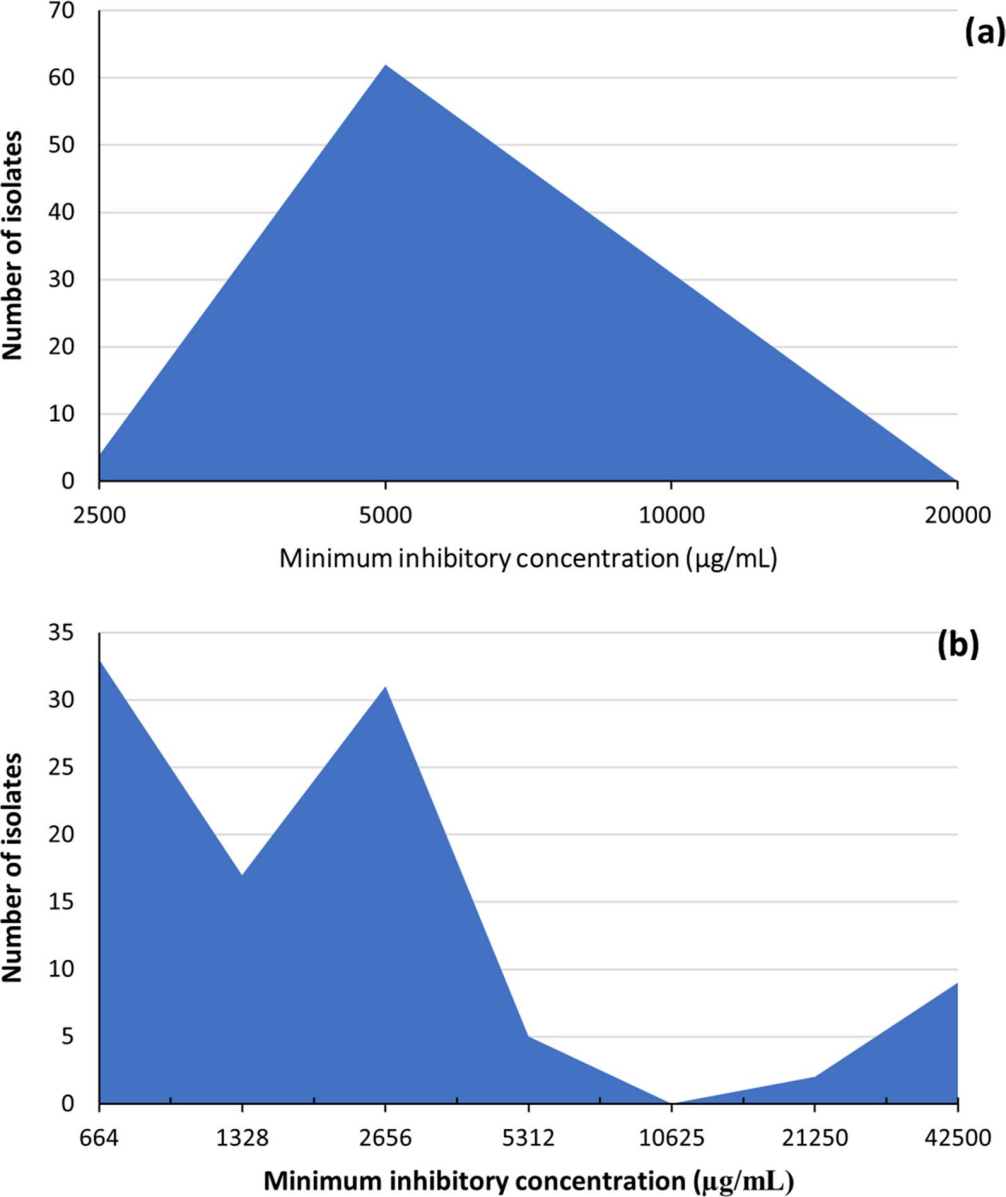
Distribution of biocides minimum inhibitory concentrations in the clinical isolates of *Staphylococcus aureus*. **a** povidone-iodine and **b** propanol-based mecetronium ethyl sulphate

The MIC of PVP-I and PBM against *S. aureus* clinical isolates (n = 97) was determined using the agar dilution method. Most of the collected isolates (62/97) had PVP-I MIC of 5000 µg/mL and 31 isolates had a MIC of 10,000 µg/mL while only 4 isolates had a MIC of 2500 µg/mL (Additional file 1). The distribution of PVP-I MIC was unimodal (Fig. 2a), and no isolates were considered resistant (the epidemiological cut-off value was 10,000 µg/mL). With PBM, most isolates had MIC ≤ 2656 µg/mL (n = 81); however, the PBM MIC had multimodal distribution (Fig. 2b). The epidemiological cut-off value of PBM was considered the MIC between the two main populations (1328 µg/mL) and 48.5% (n = 47) of the isolates were considered resistant.

The antimicrobial susceptibility of *S. aureus* clinical isolates (n = 97) was determined using Kirby-Bauer disk diffusion method. Most of the isolates (84.5%; n = 82) were MRSA. In addition, 90.7% (n = 88) of the isolates were MDR. About 99% (n = 96) of the isolates were sensitive to linezolid. There was no detectable resistance to vancomycin (Additional file 1, Fig. 3).

**Fig. 3:**
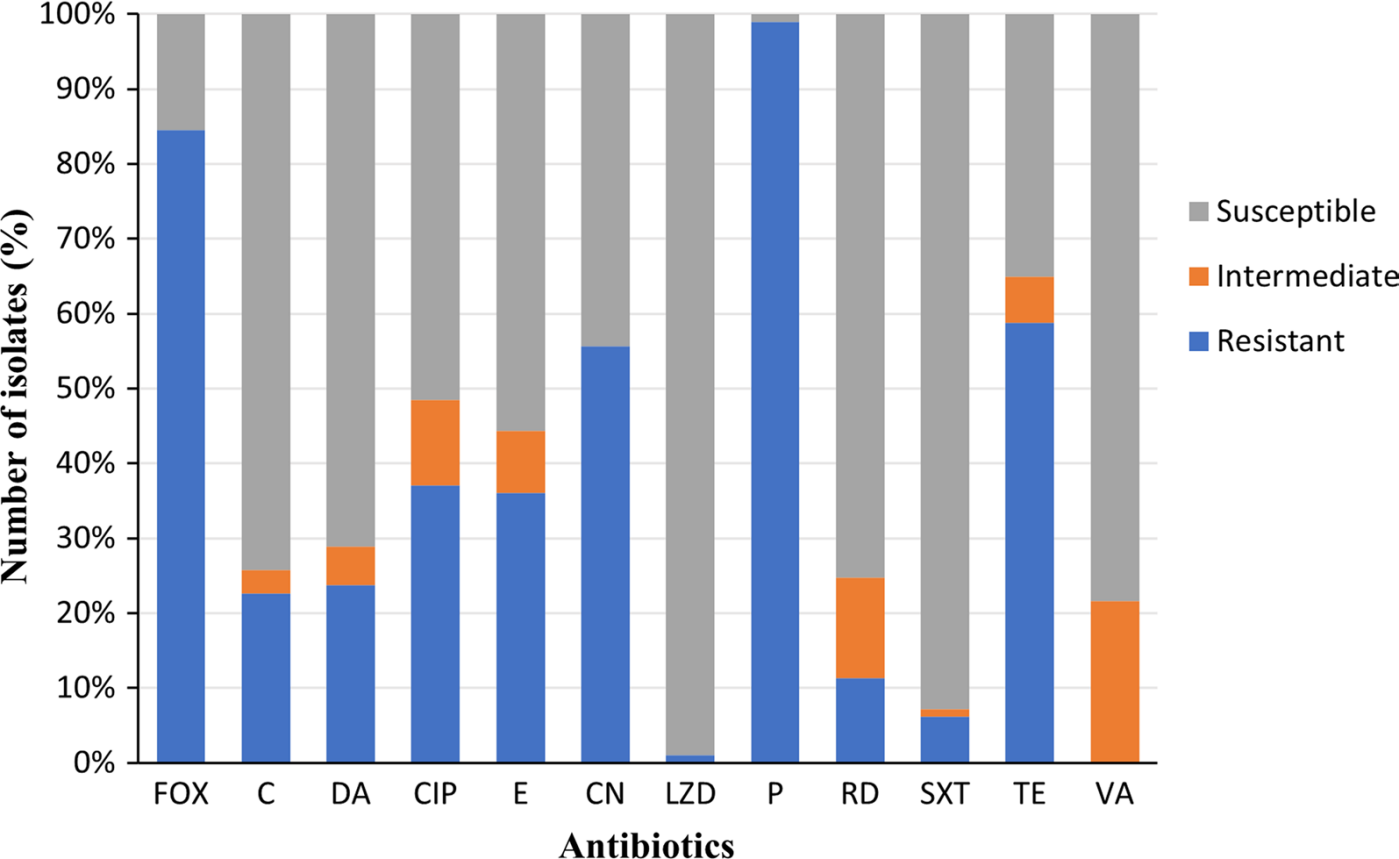
Number of clinical *Staphylococcus aureus* isolates with different antimicrobial susceptibility patterns to tested antibiotics. *C* chloramphenicol, *CIP* ciprofloxacin, *CN* gentamicin, *DA* clindamycin, *E* erythromycin, *FOX* cefoxitin, *LZD* linezolid, *P* penicillin, *RD* rifampicin, *SXT* sulfamethoxazole/trimethoprim, *TE* tetracycline, *VA* vancomycin

### Source and antibiotic susceptibility patterns of isolates in relation to PBM-resistance patterns

**Fig. 4:**
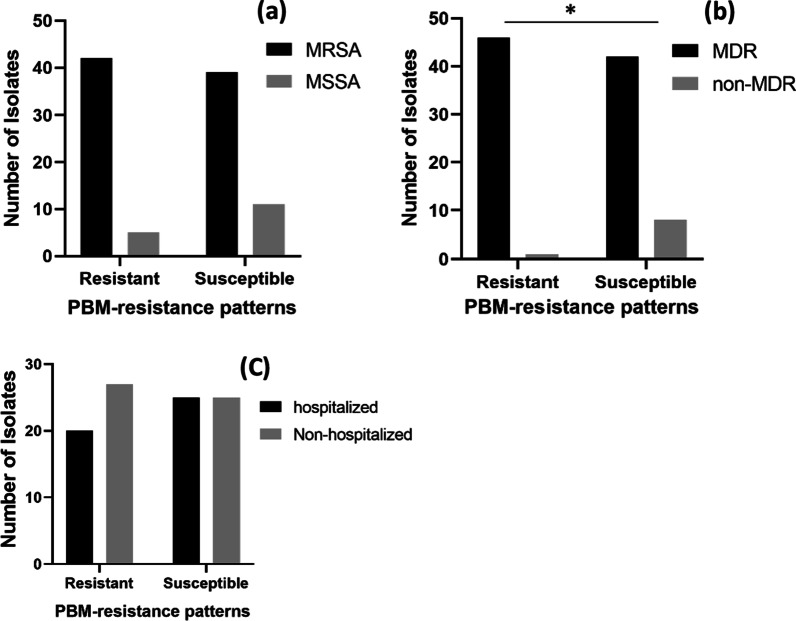
Distribution of isolates with different propanol-based mecetronium ethyl sulphate (PBM) resistance patterns. **a** among methicillin susceptible *Staphylococcus aureus* (MSSA) and methicillin resistant *Staphylococcus aureus* (MRSA), **b** among multi drug reistant (MDR) and non-MDR isolates, **(c)** among isolates from hospitalized and non-hospitalized patients. The statistical analyses were performed using Chi-square test. ^*^p < 0.05

Most PBM-resistant *S. aureus* isolates were MRSA (42/47; Fig. 4a) and MDR (46/47; Fig. 4b). No significant difference in the distribution of MRSA isolates among isolates with different PBM-susceptibility patterns (Fig. 4a) or in the distribution of PBM-resistant isolates between samples from hospitalized or non-hospitalized patients was recorded (*p* > 0.05; Fig. 4c). On the contrary, MDR isolates were more significantly detected among PBM-resistant isolates (*p* = 0.01; Fig. 4c).

### Effect of biocides’ subinhibitory concentrations on *S. aureus* virulence

**Fig. 5:**
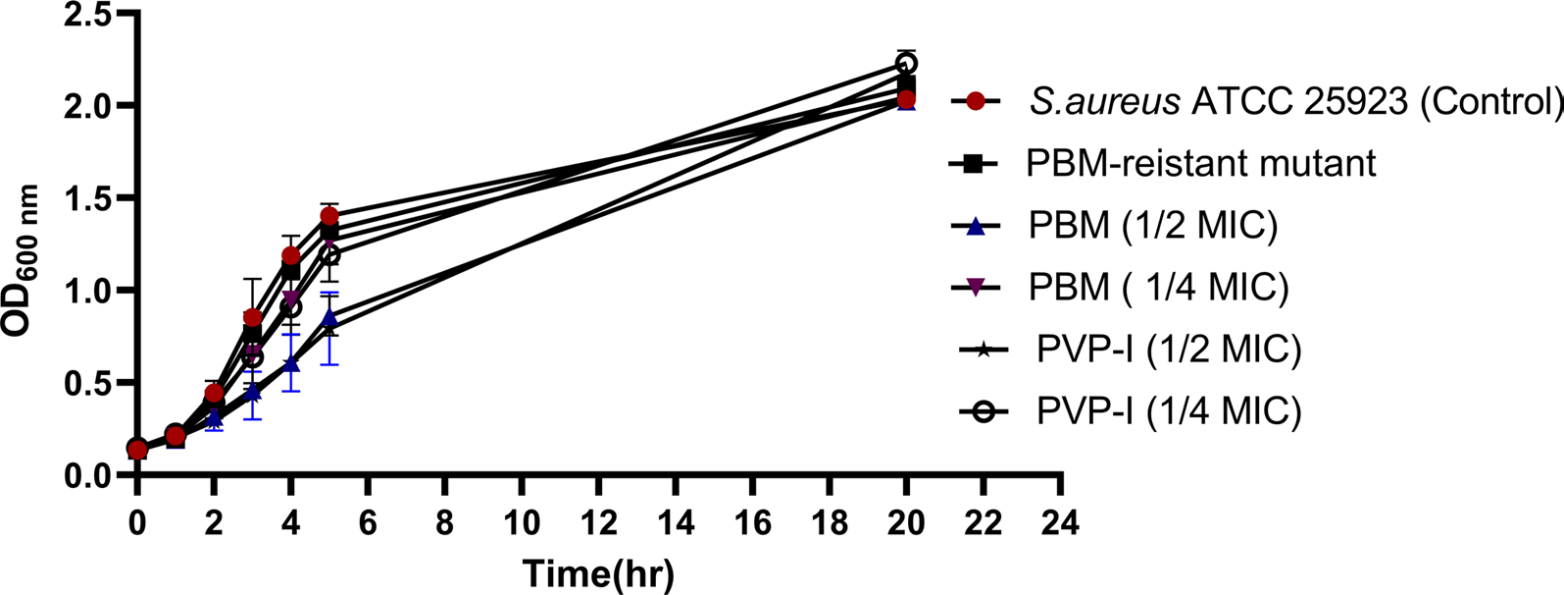
Growth patterns of parent *Staphylococcus aureus* ATCC 25923 under different conditions and of biocide-resistant mutant. Culture optical density measured hourly for the propanol based mecetronium ethyl sulphate (PBM)-resistant mutant and for the parent *S. aureus* ATCC 25923 in the absence (control) and presence of ¼ or ½ of the minimum inhibitory concentration (MIC) of either of povidone-iodine (PVP-I) or PBM. The data are represented as mean of three independent experiments. Error bars represent the standard deviation

To determine the possible effect of the subinhibitory concentrations of PVP-I and PBM on the virulence of *S. aureus* as well as to evaluate the virulence of the developed PBM-resistant mutant, the growth patterns of the developed PBM-resistant mutant and of the parent *S. aureus* strain in the absence and presence of ¼ and ½ of the MIC of either PVP-I or PBM was assessed by OD measurements of their culture at different time intervals. This confirms that any recorded difference in virulence is not due to a difference in cell density. *S. aureus* ATCC 25923 grown in the presence of ½ of MIC of PVP-I and PBM showed non-significantly reduced growth (*p* < 0.05) compared to that grown in the biocide-free medium after 5 h. However, all cultures had the same optical density after culturing for 20 h (Fig. 5).

**Fig. 6:**
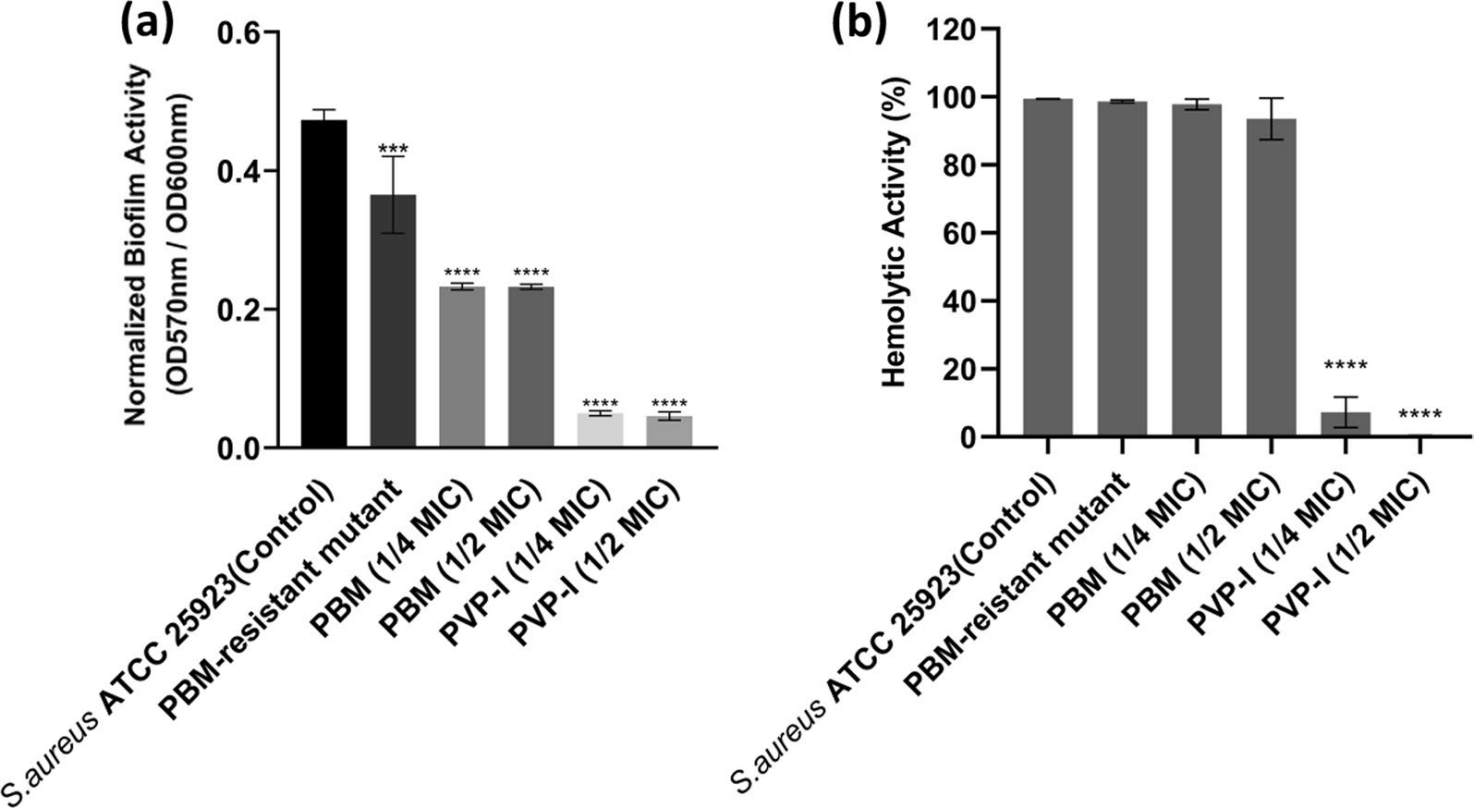
Virulence of the biocide-resistant mutant and the parent *Staphylococcus aureus* ATCC 25923 under different conditions. **a** Biofilm activity; and **b** Hemolytic activity of *S. aureus* ATCC 25923 in the absence (control) and presence of ¼ and ½ of the minimum inhibitory concentration (MIC) of povidone-iodine (PVP-I) and propanol based mecetronium ethyl sulphate (PBM) and of the PBM-resistant mutant. Error bars represent standard deviation. ***p < 0.0005, ****p < 0.0001

The biofilm formation by *S. aureus* ATCC 25923 in the presence of ¼ or ½ of the MIC of either PVP-I or PBM as well as the biofilm formation by the PBM- resistant mutant was determined using the tissue culture plate method and was compared to biofilm formation by the parent *S. aureus* ATCC 25923 in the absence of biocides. *S. aureus* ATCC 25923 grown in absence of biocides had a strong biofilm formation. The presence of ¼ or ½ of the MIC of PVP-I caused complete inhibition of biofilm formation (*p* $<$ 0.0001); however, the presence of ¼ or ½ of the MIC of PBM caused moderate biofilm formation. There was no change in biofilm activity in the PBM-resistant mutant compared to the parent strain (Fig. 6a).

Similarly, the effect of ¼ and ½ of the MIC of PVP-I and PBM on the hemolytic activity of *S. aureus* ATCC 25923 strain (hemolytic activity = 99.43%) as well as the hemolytic activity of the PBM-resistant mutant were evaluated. The presence of ½ of the MIC of PVP-I completely inhibited the alpha-hemolysin activity (hemolytic activity = 0.28%) while ¼ of the MIC of PVP-I reduced the alpha-hemolysin activity to 7.23% (*p* $<$ 0.0001). The presence of ¼ or ½ of the MIC of PBM caused a non-significant reduction in alpha-hemolysin activity (hemolytic activity was 97.81% and 93.56%, respectively). The hemolytic activity of the PBM-resistant mutant was similar to that of the parent *S. aureus* ATCC 25923 (Fig. 6b).

### Expression of *S. aureus* ATCC 25923 virulence-related genes in the presence of subinhibitory concentration of PVP-I

**Fig. 7:**
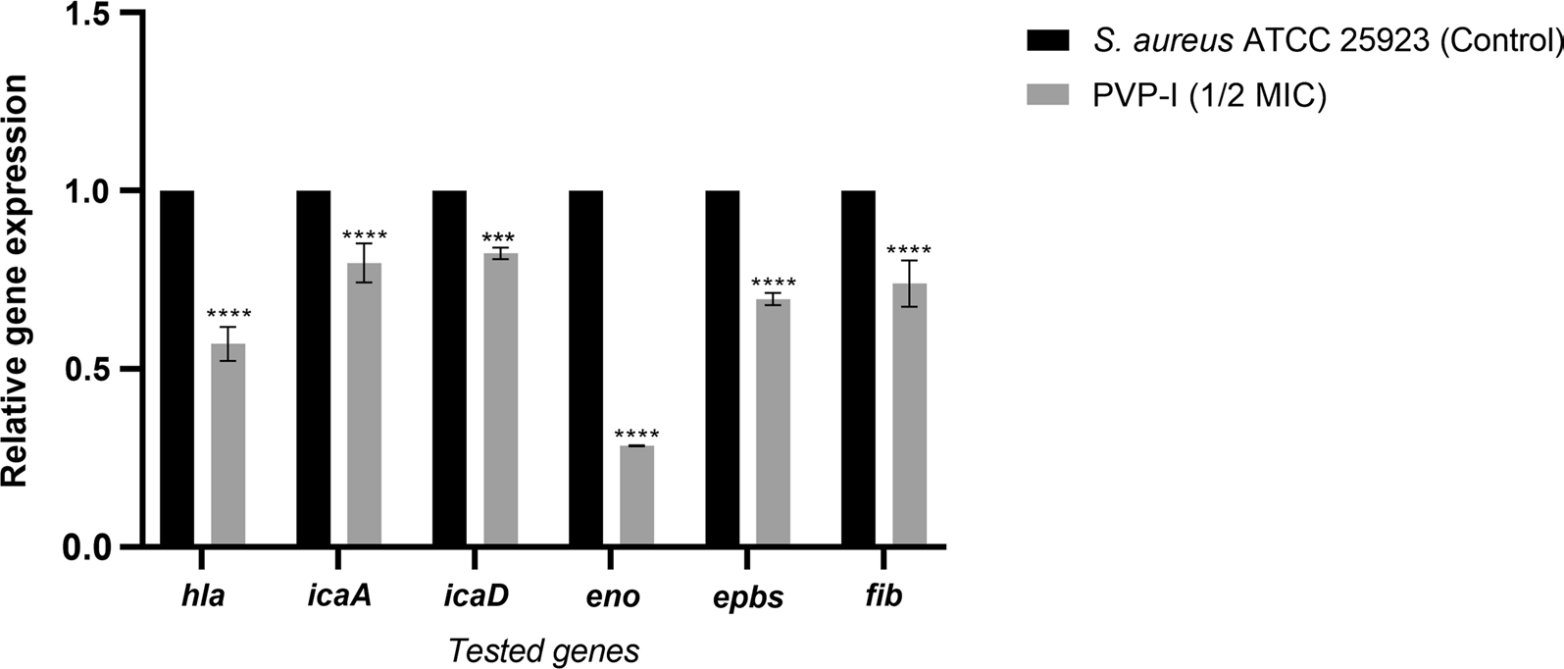
Relative expression of virulence-related genes in *Staphylococcus aureus* ATCC 25923. The relative expression of *hla, icaA, icaD, eno, epbs* and *fib* genes in *S. aureus* ATCC 25923 treated with ½ of minimum inhibitory concentration (MIC) of povidone-iodine (PVP-I) compared to that of untreated *S.* *aureus* ATCC 25923. Error bars represent the standard deviation ***p < 0.0005, ****p < 0.0001

The expression of the gene responsible for alpha-hemolysin activity (*hla*) and the genes involved in biofilm formation (*ica*A*, ica*D*, eno, epbs* and *fib*) was measured in *S. aureus* ATCC 25923 treated with ½ of MIC of PVP-I relative to that in *S. aureus* ATCC 25923 grown in absence of PVP-I. The presence of the subinhibitory concentration of PVP-I significantly reduced the transcriptional levels of all tested virulence genes (*p* $<$ 0.05; Fig. 7).

## Discussion

Besides the improper use of antibiotics that has led to the emergence of antimicrobial resistance, concerns have been raised on the role of non-antibiotic antimicrobials in the evolution and selection of antimicrobial-resistant strains [36]. Some reports are available on the role of indiscriminate use or repeated exposure to subinhibitory concentrations of biocides in antibiotics cross-resistance [3, 7].

In this study, the possible development of *S. aureus* resistance to PVP-I and PBM by repeated exposure was evaluated. No resistance was developed to PVP-I after ten consecutive passages of *S. aureus* ATCC 25923 in subinhibitory PVP-I concentration (1/2 of MIC). Similarly, Wiegand and his colleagues have reported that PVP-I didn’t trigger resistance in *S. aureus* ATCC 6538 [37]*.* Also, no resistance to PVP-I was developed in *P. aeruginosa, Escherichia coli, Klebsiella aerogenes,* and *Serratia marcescens* after 20 serial passages [38]. Further studies are required to test the possibility of resistance development to PVP-I after more than ten consecutive passages.

On the contrary, the growth of *S. aureus* ATCC 25923 in increasing subinhibitory concentrations of PBM resulted in the generation of a stable PBM-resistant mutant with a 128-fold increase in PBM MIC. This was not the case with MDR *P. aeruginosa* isolates that failed to adapt to PBM after four passages [39]. No reports are available on resistance development to PBM in *S. aureus* or in other microbial species, by repeated exposure; however, reports are available on the development of resistance in different *Staphylococcus* spp. to triclosan and chlorophene by repeated exposure [40, 41].

The development of resistant strains in the hospital environment is very dangerous, as the hospital environment represents a high-risk community where the evolution of clinically dangerous clones starts sequentially and expands to the community population [42]. PVP-I is extensively used in all healthcare settings; in the hospital environment, subinhibitory concentrations of PVP-I may be clinically present due to the dilution of the antiseptic with tissue fluids, furthermore, residues of diluted PVP-I may remain on the skin after use [21, 40, 43]. Similarly, subinhibitory concentrations of PBM may also be present.

Our previous results were further confirmed by the results recorded for the collected *S. aureus* clinical isolates, where no isolates were resistant to PVP-I while about 47.5% of the isolates were PBM-resistant. This confirms the matching of the results from laboratory studies and the real-life situation regarding resistance development to PVP-I and PBM. Exposure to PBM but not PVP-I caused the development of resistance, where our isolates were collected from the hospital during the use of PVP-I and PBM for antisepsis.

The multimodal distribution of PBM MIC indicates the presence of different populations regarding their susceptibility to PBM similar to antibiotics MIC distribution that is bimodal or multimodal in most cases [32]. The lack of significant difference between the number of PBM-resistant strains in isolates from hospitalized and non-hospitalized patients indicates the spread of PBM-resistant clones not only in hospitals but also in the community. This may be due to the widespread use of PBM as hand antiseptic inside and outside the hospitals.

What worsens the condition is the reported cross-resistance to some antibiotics in the developed PBM-resistant mutant. PBM-resistant mutant acquired resistance to penicillin, cefoxitin, and ciprofloxacin. Similarly, amoxicillin resistance was reported in PBM-treated *E. coli* [44]. A previous report on decreased penicillin susceptibility by exposure of *S. aureus* ATCC 25923 to octenidine dihydrochloride and chlorhexidine is available [9]. The development of antibiotic cross-resistance in PBM-resistant strains is further confirmed by the significantly higher number of MDR-resistant isolates that are PBM-resistant. However, we didn’t find any significant difference between the number of MRSA isolates among different PBM-susceptibility patterns. Similarly, no significant difference was reported in the incidence of triclosan or chlorhexidine resistance among MSSA or MRSA strains [45]. Contrary, Curiao, and colleagues have reported increased susceptibility to different antimicrobial agents in benzalkonium chloride-adapted *Salmonella enterica* Typhimurium, as a fitness cost [46].

This is the first report on the distribution of PBM MIC in a natural isolates. As described earlier, the collected isolates represent natural isolates that were not subjected to multiple subcultures in the laboratory. Their resistance pattern is similar to that reported in other countries, where most of the tested *S. aureus* clinical isolates (84.5%) were MRSA. Similar rates of MRSA (79.6%) were reported in Egypt [47, 48] and other countries such as Italy, Portugal, and France [49, 50]. The predominance of MDR was recorded elsewhere [47, 51, 52]. In addition, all the tested isolates were resistant to penicillin. Similarly, ElSayed et al. reported that 96% of their isolates were penicillin-resistant [53]. Therefore, testing for PBM resistance and MIC distribution in other areas is highly recommended to detect the possible emergence of resistant strains and implement protocols to limit their spread.

Biocides’ mechanism of action isn’t completely clear because of their ability to act concurrently on multiple sites and their resistance and cross-resistance to antimicrobial agents are usually mediated by non-specific mechanisms [11, 54]. Such resistance in *S. aureus* can be developed due to induction of multidrug efflux pumps, alteration of target sites, or cell wall changes [55], where in our study two different classes of antibiotics were affected; cell wall active agents (penicillin and cefoxitin) and fluoroquinolones (ciprofloxacin) confirming the possibility of a nonspecific resistance mechanism. In benzalkonium chloride-adapted *E. coli*, overexpression of genes encoding efflux pumps was confirmed in addition to the downregulation of genes encoding membrane porins [56]. In *P. aeruginosa* chlorhexidine-adapted strains, cross-resistance was suggested to be a result of efflux pump overexpression, porin loss, or change in membrane permeability [11]. However, in *Acinetobacter baumannii*, no cross-resistance was recorded between PBM or ethanol and antimicrobials [57].

*S. aureus* can cause a wide range of critical life-threatening infections in humans; such ability is related to the expression of vast arrays of virulence factors that directly impact disease pathogenesis and severity and contribute to antimicrobial resistance and treatment failure [58]. Inhibition of virulence is considered among the possible alternatives that control infections, especially in the case of MDR organisms [20]. Inhibition of *S. aureus* virulence was successfully capable of controlling the infection in a murine model [59].

Biofilm formation is one of the main virulence factors that notoriously complicate the treatment of *S. aureus* infections. The prescence of subinhibitory concentrations of PVP-I (¼ and ½ of MIC) completely abolished biofilm formation in *S. aureus* ATCC 25923. Similar results about the inhibition of biofilm formation by subinhibitory concentrations of PVP-I in *S. aureus* and other pathogens (*K. pneumoniae*, *P. aeruginosa,* and *Candida albicans*) were reported previously [17, 60, 61]. Similarly, subinhibitory concentrations of PBM were able to reduce biofilm production, but to a lesser extent. No reports are available on the effect of subinhibitory concentrations of PBM on biofilm formation in *S. aureus* or other pathogens. Sublethal doses of benzalkonium chloride and trisodium phosphate were able to slightly inhibit biofilm formation in MRSA strain [62]. Also, chlorhexidine and glutaraldehyde inhibited biofilm formation in *P. aeruginosa* to a lesser extent. However, other studies reported the induction of biofilm formation in *S. aureus* and *Staphylococcus epidermidis* by exposure to different concentrations of some alcoholic disinfectants (ethanol, n-propanol, and isopropyl alcohol) and sodium hypochlorite [62–65]. This difference in the effect of PBM and alcoholic disinfectants on biofilm formation may be due to the presence of mecetronium ethyl sulphate as a component of PBM.

Alpha-hemolysin is another important virulence protein that contributes to *S. aureus* pathogenesis. *S. aureus* alpha-hemolysin mediates the death of many cells including erythrocytes and immunological cells thus playing a vital role in soft tissue and skin infections [16]. PVP-I subinhibitory concentrations (¼ and ½ of the MIC) dramatically reduced the hemolytic activity of *S. aureus* ATCC 25923 to 7% and 0.28%, respectively (*p* $<$ 0.0001). This comes in agreement with other studies that reported the ability of PVP-I to suppress hemolysin production in *S. aureus* and *E. coli* [66]. The role of PVP-I in the inhibition of several bacterial exotoxins was reported previously [66]. Unlike PVP-I, subinhibitory concentrations of PBM didn’t affect the hemolysin activity.

The levels of biofilm formation and hemolysin activity in the PBM-resistant mutant were similar to that of the parent *S. aureus* ATCC 25923. Other studies reported controversy results regarding the virulence of different biocide-resistant strains. Triclosan-adapted *S. aureus* had reduced biofilm formation and hemolysin activity [67, 68]. However, exposure of pathogenic *E. coli* to biocides as benzalkonium chloride and triclosan caused an increase in biofilm formation [69]. Similarly, Forbes and colleagues recorded an increased biofilm formation in benzalkonium chloride adapted-*E. coli* [56].

The inhibition of virulence (biofilm formation and hemolysin activity) in *S. aureus* by subinhibitory PVP-I concentrations was found to be a result of an effect on the expression of genes encoding alpha-hemolysin (*hla*) or involved in biofilm formation (*ebps*, *eno*, *fib*, *ica*A, and *ica*D), where the expression level of the aforementioned genes was significantly reduced in presence of ½ of MIC of PVP-I. Similarly, Oduwole et al. reported that the subinhibitory concentration of betadine reduced the transcription of *ica*ADBC operon in *S. aureus* RN 4220 and *S. epidermidis* 1457 [17].

Further studies on the exact possible mechanisms of PBM resistance in *S. aureus* strains are highly recommended. The effect of subinhibitory concentrations of PVP-I and PBM on biofilm formation and hemolysin activity in pathogens other than *S. aureus* needs to be further elucidated.

## Conclusion

We were able to study the effect of two main hospital-used antiseptics on two important aspects of the evolution of clinically dangerous clones of *S. aureus* in a high-risk community. PVP-I exposure didn’t induce the emergence of resistant *S. aureus* strains and can inhibit *S. aureus* biofilm formation and hemolysin activity which is highly advantageous in healthcare settings to control the emergence of resistant strains. This highlights the superiority of PVP-I as a pre-operative antiseptic and a promising effective agent for the management of nosocomial infections and in limiting the evolution of dangerous clones in the hospital community. However, repeated exposure of *S. aureus* to PBM in healthcare settings may contribute to PBM resistance development and the emergence of antibiotics cross-resistance. Therefore, special protocols need to be implemented while using PBM, where PBM is one of the most used hand antiseptics inside and outside the hospital community.

## Supplementary Information

**Additional file 1.** **Table S1**: The minimum inhibitory concentration (MIC) of PVP-I and PBM, antimicrobial susceptibility profile and the corresponding resistance phenotype of *S.* *aureus* clinical isolates.

## Data Availability

All data generated or analyzed during this study are included in this published article and its additional information file.

## Abbreviations

CDC: Centers for Disease Control and Prevention
CLSI: Clinical and Laboratory Standards Institute
FDA: Food and Drug Administration
MDR: Multidrug resistant
MH: Muller Hinton
MIC: Minimum inhibitory concentration
MRSA: Methicillin resistant *Staphylococcus aureus*
MSCRAMMs: Microbial surface components recognizing adhesive matrix molecules
MSSA: Methicillin susceptible *Staphylococcus* *aureus*
NB: Nutrient broth
OD: Optical density
ODc: Optical density cut-off value
PBM: Propanol-based mecetronium ethyl sulphate
PBS: Phosphate buffered saline
PIA: Polysaccharide intracellular adhesion
PVP-I: Povidone-iodine
qRT-PCR: Quantitative real-time polymerase chain reaction
TSB: Tryptone soya broth
